# Early Biological Response to Poly(ε-caprolactone) PCL—Bioactive Glass Composites Obtained by 3D Printing as Bone Substitutes

**DOI:** 10.3390/polym17162229

**Published:** 2025-08-15

**Authors:** Alessandro Mosca Balma, Riccardo Pedraza, Ilaria Roato, Clarissa Orrico, Sara Meinardi, Stefano Bertinetti, Tullio Genova, Giovanna Gautier di Confiengo, Maria Giulia Faga, Donatella Duraccio, Giulio Malucelli, Marta Miola, Enrica Verné, Federico Mussano

**Affiliations:** 1 Bone and Dental Bioengineering Laboratory, CIR Dental School, Department of Surgical Sciences, University of Turin, 10126 Turin, Italy; alessandro.moscabalma@unito.it (A.M.B.); or riccardo.pedraza@stems.cnr.it (R.P.); ilaria.roato@unito.it (I.R.); or clarissa.orrico@polito.it (C.O.); 2 Department of Mechanical and Aerospace Engineering, Politecnico di Torino, Corso Duca degli Abruzzi 24, 10129 Turin, Italy; 3 Institute of Sciences and Technologies for Sustainable Energy and Mobility, National Council of Research, Strada delle Cacce 73, 10135 Turin, Italy; giovanna.gautier@stems.cnr.it (G.G.d.C.); mariagiulia.faga@stems.cnr.it (M.G.F.); donatella.duraccio@stems.cnr.it (D.D.); 4 Department of Life Sciences and Systems Biology, University of Torino, Via Accademia Albertina 13, 10123 Torino, Italy; sara.meinardi@edu.unito.it (S.M.); tullio.genova@unito.it (T.G.); 5 Department of Chemistry, University of Torino, Via P. Giuria 7, 10125 Torino, Italy; stefano.bertinetti@unito.it; 6 Department of Applied Science and Technology, Politecnico di Torino, Viale T. Michel 5, 15121 Alessandria, Italy; giulio.malucelli@polito.it; 7 Department of Applied Science and Technology, Politecnico di Torino, Corso Duca degli Abruzzi 24, 10129 Turin, Italy; marta.miola@polito.it (M.M.); enrica.verne@polito.it (E.V.)

**Keywords:** Poly(ε-caprolactone), bioactive glass, copper-doped, solvent casting, 3D printing, cell viability, early cell response, adipose-derived mesenchymal stem cells

## Abstract

The increasing demand for smart bone substitutes has boosted the implementation of biomaterials possibly endowed with both pro-osteogenic and pro-angiogenic capabilities, among which bioactive glasses hold great potential. Hence, two Poly(ε-caprolactone) (PCL)-based composites were loaded at 10 wt.%, with either pristine (SBA3) or copper-doped (SBA3_Cu) silica-based bioactive glasses, through a solvent casting method with chloroform. Neat PCL was used as a control. Samples produced by 3D printing underwent SEM and EDX analyses, and the following were measured: tensile strength and hardness, surface roughness, ion release through ICP-OES, surface free energy, and optical contact angle. Adipose-derived mesenchymal stem cells (ASCs) and human microvascular endothelial cells (HMEC-1) were used to test the biocompatibility of the materials through cell adhesion, spreading, and viability assays. A significant improvement in tensile strength and hardness was observed especially with Cu-doped composites. Both SBA3 and SBA3_Cu added to the PCL favored the early adhesion and the proliferation of HMEC-1 after 3 and 7 days, while ASCs proliferated significantly the most on the SBA-containing composite, at all the time points. Cellular morphology analysis highlighted interesting adaptation patterns to the samples. Further biological characterizations are needed to understand thoroughly how specific bioactive glasses may interact with different cellular types.

## 1. Introduction

Engineered scaffolds for tissue regeneration play a fundamental role in the field of tissue engineering. Biocompatibility, an open and interconnected porosity, appropriate mechanical properties to the host tissue, and bioactivity are some of the characteristics that a scaffold must possess to integrate with the receiving site [1]. Techniques that exploit artificial scaffolds for bone replacement (alloplast) could be adopted for personalized medicine [2], and, from an economic point of view, are more suitable compared to bone extracted from other species (xenografts) or compatible human donors (allografts). Further, they avoid the risk of morbidity in the donor site of tissue obtained from the same patient (autograft) [3,4].

Various materials and techniques have been studied and adopted to create effective scaffolds for regenerative medicine. Additive manufacturing (AM), and especially 3D printing, is one of the most studied and promising technology for scaffold development. Among all printing techniques, fused deposition modeling (FDM) is a versatile solution for several materials including synthetic polymers like Polylactic acid (PLA), Poly (lactide-co-glycolide) (PLGA) Polyamides, and Poly(ε-caprolactone) (PCL) [5,6]. In this context, PCL is a synthetic and bioresorbable aliphatic polyester, gaining attention as a promising material among polymers for bone tissue engineering (BTE) applications [7]; PCL has been suggested for the creation of composite materials through the incorporation of various fillers such as alumina-toughened zirconia (ATZ) [8], SiO_2_ [9], and CaO [10].

Bioactive glasses are a family of materials (such as 45S5 [11], S53P4 [12], 70S30C [13]) with a wide range of applications: in particular, they are extensively studied for dental and maxillofacial uses [14], due to their biodegradability, biocompatibility, osteoconductive properties, and ability to enhance bone regeneration [15]. In restorative dentistry, bioactive glass fillers and coatings improve properties like antimicrobial activity, hardness, and remineralization, making them valuable additives in materials such as glass ionomer cements and zirconia-based implants [16]. Recently, bioactive composite scaffolds consisting of a bioresorbable matrix and a bioactive inorganic phase, including bioactive glasses [17], hydroxyapatite [18], and calcium phosphates [19], have attracted great interest in the biomedical community [20,21,22,23,24].

Among the bioactive inorganic phases, bioactive silica-based glasses are widely investigated in tissue regeneration and especially for bone repair, thanks to their ability to stimulate cell adhesion, proliferation, and differentiation, triggered by the release of specific ions [25,26]. Furthermore, the introduction of special elements in the glass composition can impart therapeutic properties such as antibacterial (e.g., Ag, Cu, Zn [27]), pro-angiogenic (e.g., Cu, Co [28]), or antioxidant (e.g., Te, Ce [29,30]) effects. The literature reports several studies on PCL-based composite materials (mainly fibrous matrices) containing bioactive glasses doped with different elements [31,32]. In this work, PCL was used as a polymeric matrix for the preparation of two composites, characterized by the presence of two different formulations of the same silica-based bioactive glass, namely, the pristine one (SBA3) and the one doped with copper ions (SBA3_Cu). These two different bioactive glass-PCL compounds, along with neat PCL as a control, were studied through their physical, mechanical, and early biological properties, tested with two different cell types representing standards for pre-osteoblast and vascular tissue formation in in vitro models: mesenchymal stem cells derived from adipose tissue ASC52hTert (ASC) and Human Microvascular Endothelial Cells (HMEC-1).

With these premises, the authors’ purpose was to produce and characterize PCL-SBA solvent-casted and printed samples [8], potentially useful in the fabrication of bone substitutes, hence improving the current state of alloplasts for bone regeneration.

## 2. Materials and Methods

### 2.1. Sample Preparation

An ester-terminated polycaprolactone (CAS-n 24980-41-4) matrix (CELLINK PCL TP-60505, Bico Group, Gothenburg, Sweden) was loaded with two bioactive glasses at 10 wt.%, namely PCL/SBA3 90/10 and PCL/SBA3_Cu 90/10, through solvent casting with Chloroform (CHCl_3_, CARLO ERBA Reagents s.r.l., Cornaredo, Italy), as previously described [8]. SBA3 glass powders < 20 μm (48% SiO_2_, 26% Na_2_O, 22% CaO, 3% P_2_O_5_, 0.43% B_2_O_3_, 0.57% Al_2_O_3_—mol%) were produced by the melting and quenching technique, followed by milling and sieving processes as reported in [33]. Subsequently, copper was introduced in SBA3 powders through an ion-exchange process in copper acetate solution 0.01 M for 1 h at 37 °C [33]. These compositions were selected for their proven bioactivity and the antibacterial properties (SBA3_Cu) [34]. Neat PCL was considered a control. The composition of the investigated samples is reported in Table 1.

Three-dimensional planar samples were printed using the thermoplastic pneumatic printhead of BIO X 3D bioprinter (CELLINK Bico Group, Gothenburg, Sweden). A square base with dimensions of 15 mm × 15 mm, 0.65 mm thick, and 100% infill was printed directly onto the glass surface of a Petri dish to ensure the smoothest possible support surface. Cylindrical disks were produced by cutting the 3D-printed square structure using a 6 mm biopsy punch. These disks underwent protein adsorption, ion release analysis, cell adhesion, cell spreading, cell viability assays, SEM analysis, and contact angle evaluation. Specimens measuring 8 mm × 8 mm × 1 mm and 50 mm × 3 mm × 1 mm were printed, respectively, for nanoindentation and mechanical tests. The print-bed was maintained at 30 °C with the clean chamber fan activated. All materials were extruded at a printhead temperature of 115 °C. A 0.4 mm diameter nozzle was used, with an applied pressure of 190 kPa and a printing speed of 2 mm/s.

### 2.2. Microscopy and EDX Spectroscopy

The morphology of the different surfaces was studied using a Scanning Electron Microscope (Phenom XL G2 Desktop SEM, Thermo Fisher Scientific, Waltham, MA, USA). Samples were washed sequentially in distilled water, and in a 70 vol.% ethanol/water solution, before ultrasonic cleaning in absolute ethanol for 20 min, followed by air-drying under a chemical hood. Before SEM analysis, a coating with a conductive layer of gold was applied. The instrument settings were 10 kV of source potential in MAP configuration, with a BSD detector with a magnification of 1000×.

### 2.3. Mechanical Properties

Tensile tests were performed using an Instron 5966 dynamometer (Norwood, MA, USA) at room temperature. The size of the samples was 50 × 3 × 1 mm^3^, with a gauge length set by positioning the clamps 2 cm apart. The testing procedure involved an initial loading rate of 1 mm/min up to 0.2% strain, followed by a rate of 30 mm/min until break. The parameters recorded included Young’s modulus (*E*), elongation at yield (ε_y_), strength at yield (σ_y_) tensile strength (σ_b_), and elongation at break (ε_b_). The mechanical properties were determined as the average values from ten independent tests performed on each sample group.

Hardness was assessed with a Vickers indenter by a FISCHERSCOPE HM 2000 XYm (Helmut Fischer GmbH, Sindelfingen, Germany). Ten repetitions were carried out for each group of samples on specimens measuring 8 × 8 × 1 mm^3^. Vickers hardness (HV) and Young’s moduli (EiT) values were calculated through Load–depth curves, as previously described [35].

### 2.4. Roughness

The surface roughness was evaluated using the integrated tool for 3D Roughness Reconstruction (3DRR) (Phenom XL G2 Desktop SEM, Thermo Fisher Scientific, Waltham, MA, USA). S_a_ parameter was measured, keeping the area of the measurement at 120 µm × 120 µm and using a First Order Correction (FOC) filter. With this technique, linear roughness parameters of Arithmetical Mean Height (R_a_) and Maximum Height (R_z_) were also measured. All the measurements were performed on the smoothest surface of the samples printed directly on a Petri dish.

### 2.5. Contact Angle and Surface Free Energy Evaluation

Surface wettability was assessed through Biolin Scientific Theta Lite Optical Tensiometer (Stockolm, Sweden) using double-distilled water (ddH_2_O) and di-iodomethane (CH_2_I_2_, CAS-No.: 75-11-6, Sigma-Aldrich, St. Louis, MO, USA). The contact angle was evaluated by the sessile drop method, as described elsewhere [36].

The Owens–Wendt–Rabel–Kaelbel (OWRK) method was applied to calculate the value of surface free energy following the method proposed by Waldner et al. [37], as reported previously [35]. Total (γ), polar (γ^P^), and dispersive (γ^D^) components were calculated by simple linear regression, considering the properties of ddH_2_O and CH_2_I_2,_ to perform the interpolation (Table 2).

### 2.6. Ion Release Analysis

To analyze the ion release, the samples were maintained in alpha-MEM in a cell culture incubator, for 3, 7, and 15 days. The supernatants were collected and analyzed using an ICP-OES (iCAP Pro X, Thermo Fisher Scientific, Waltham, MA, USA). The concentration of Al, P, Ca, B, Si, and Cu in the solutions were determined by external calibration method, preparing multi-elemental standards solution at desired concentration from single elemental stock solutions (1000 mg/L, Merck, Darmstadt, Germany).

### 2.7. Protein Adsorption

To measure the amount of protein adsorbed onto the samples, Bovine Serum Albumin (BSA, CAS No.: 9048-46-8, Sigma-Aldrich, St. Louis, MO, USA) was prepared at a 5% (*w*/*v*) concentration in Phosphate-Buffered Saline (PBS, Euroclone, Pero, Italy). The samples were incubated with this solution at 37 °C for 20 min. Following incubation, they were rinsed twice with PBS. The total protein content was subsequently quantified using the Pierce™ BCA Protein Assay Kit (Life Technologies, Carlsbad, CA, USA) following the manufacturer’s protocol.

### 2.8. Cell Culture

ASC52hTert cell line, adipose-derived mesenchymal cells (ASCs) (ATCC, Manassas, VA, USA), and human microvascular endothelial cells (HMEC-1) (CLS, Cell Lines Service GmbH, Eppelheim, Germany) were used to investigate the biocompatibility of the different printed samples. ASCs and HMEC-1 were expanded, respectively, in ASC medium (Mesenchymal Stem Cell Basal Medium (ATCC PCS-500-030) with a Mesenchymal Stem Cell Growth Kit (ATCC PCS-500-040)), and in MCDB131 medium (Life Technologies, Carlsbad, CA, USA) + 1% L-Glutamine, 10% fetal bovine serum (FBS), 1% Penicillin (100 U/mL)-Streptomycin (100 μg/mL) (Life Technologies, Carlsbad, CA, USA), 1 ug/mL Hydrocortisone and 10 ng/mL EGF (Merck KGaA, Darmstadt, Germany), at 37 °C in 5% CO_2_ atmosphere. To perform the following experiments, ASCs were seeded in alpha-MEM with 10% fetal bovine serum (FBS), 1% Penicillin (100 U/mL)-Streptomycin (100 μg/mL) (Life Technologies, Carlsbad, CA, USA), and HMEC-1 were kept in their expansion medium.

#### 2.8.1. Cell Adhesion and Spreading

To assess cell adhesion, cells were seeded at a density of 8000 cells per well on the specimens, in 96-well culture plates, and incubated for 20 min. Afterward, they were fixed with a 4% paraformaldehyde solution for 8 min, rinsed with PBS, and stained with DAPI to mark to the nuclei (Merck KGaA, Darmstadt, Germany).

To assess cell spreading, an equal number of cells were cultured for 24 h, then fixed and stained with Phalloidin (Cell Signalling Technology, Danvers, MA, USA) to label the cytoskeleton, and DAPI for the nuclei. Images were captured using a Nikon Eclipse Ti-E microscope equipped with Nikon Plan 20X/0.75 and 10X/0.10 objectives. Nuclei were quantified using the ‘Analyze Particles’ function in ImageJ software (U.S. National Institutes of Health, Bethesda, MD, USA, http://imagej.nih.gov/ij/, accessed on 30 June 2025). Four high-magnification images were acquired for each sample and were analyzed using the Cellpose segmentation algorithm (Cellpose-SAM) [38], which was pre-trained with similar datasets to accurately outline individual cells. A series of morphological features were then measured for each identified cell using the Set Measurements tool in Fiji/ImageJ, including area, perimeter, BFE aspect ratio, and circularity. The resulting data were analyzed and visualized using MATLAB (R2024a; The MathWorks, Inc., Natick, MA, USA).

#### 2.8.2. Cell Viability

ASCs and HMEC-1 were plated on the samples at a density of 10,000 cells/well in 96-well culture plates. Cell viability was assessed through relative light unit (RLU) after 1, 3, and 7 days of culture in vitro, using Cell Titer GLO kit (Promega, Madison, WI, USA), according to the manufacturer’s protocol.

### 2.9. Statistical Analysis

If not otherwise stated, statistical analyses were conducted using STATA software (version 18.0; StataCorp, College Station, TX, USA). A one-way ANOVA was used to assess variance differences among groups across various time points, followed by a Bonferroni post hoc correction to identify which groups showed statistically significant differences. Repeated *t*-tests were applied to analyze shape descriptors related to cell morphology. A significance threshold of α = 0.05 was used for all tests [39].

## 3. Results

### 3.1. Scanning Electron Microscopy and Elemental Analyses of the Composites

**Figure 1:**
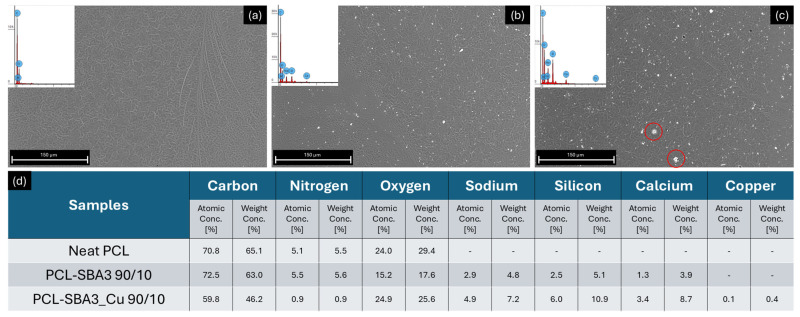
Scanning electron micrographs of materials: (**a**) neat PCL, (**b**) PCL/SBA3 90/10, (**c**) PCL/SBA3_Cu 90/10, at 1000× magnification (red circles indicate filler aggregates). (**d**) Table of detected elements on the surfaces of the samples through EDX.

PCL/SBA3 90/10 (Figure 1b) and PCL/SBA3_Cu 90/10 (Figure 1c) exhibited similar surface morphologies. The filler particles were well dispersed and evenly distributed in both composite materials. No significant bioactive glass aggregates were visible in PCL/SBA3 90/10. However, in PCL/SBA3_Cu 90/10, small filler aggregates were observed, likely due to the higher electrostatic charge of the copper-doped particles (highlighted in red circles), which tended to cluster and form nucleation centers. Neat PCL (Figure 1a) displayed a clean and uniform surface, with minor imperfections likely caused by the printing process.

EDX spectroscopy performed on the sample surfaces (Figure 1d) confirmed the composition of the composite materials. In neat PCL, the only detected elements were carbon, nitrogen, and oxygen, which form the molecular backbone of the polymer matrix. Regarding the bioactive glass components, spectroscopy of PCL/SBA3 90/10 revealed all major filler elements, such as sodium, silicon, and calcium. In the PCL/SBA3_Cu 90/10 sample, in addition to these elements, copper, which was introduced by ion-exchange process, was also detected. The ion-exchange technique allows the introduction of a limited amount of copper into the glass composition, and only at the surface level to avoid cytotoxic effects. The % of the glass elements (sodium, silicon, and calcium) were detected in higher amounts in PCL/SBA3_Cu 90/10 compared to PCL/SBA3 90/10, probably due to the higher agglomeration and surface exposition (Figure 1c), as evidenced also by Piatti et al. [21].

### 3.2. Mechanical Characterization of the Composites

**Figure 2:**
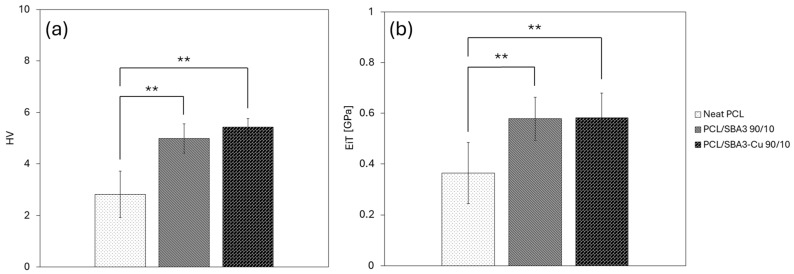
HV (**a**) and EiT (**b**) hardness measures performed on each sample (significance: (**) for *p* < 0.01).

The mechanical properties of the scaffolds, including Young’s modulus (*E*), elongation (ε_y_) and strength at yield (σ_y_), tensile strength (σ_b_), and elongation at break (ε_b_), are presented in Table 3. The introduction of SBA3 into PCL resulted in a statistically significant enhancement of Young’s modulus. Interestingly, a combination of SBA3 particles and Cu allowed us to achieve nearly 7-fold higher values of Young’s modulus compared to neat PCL: this finding is in agreement with the hardness measurements below (Figure 2). The composite scaffolds showed an increase in the elongation at yield, while yield strength and the break parameters slightly decreased when compared with neat PCL. However, the composites retained the ductile and yielding behavior characteristic of the neat polymer.

Figure 2 shows Vickers hardness (HV) and Young’s modulus results (EiT). HV values of PCL significantly increased with the addition of bioglass (*p* < 0.01) and almost doubled with the addition of Cu-doped bioglass (PCL/SBA3_Cu 90/10). Furthermore, the standard deviation decreased with the addition of both SBA3 and SBA3_Cu. The same trend was observed for Young’s modulus, where a relevant increase in EiT value was observed for PCL/SBA3 90/10 and PCL/SBA3_Cu 90/10 (*p* < 0.01) samples, and a lower dispersion of data as well.

### 3.3. Roughness

As reported in Table 4, the composite materials showed a slightly decreased areal average roughness (S_a_) compared to the neat polymer; indeed, only PCL/SBA3_Cu 90/10 differed significantly from PCL (*p* = 0.048). The addition of the fillers significantly decreased the R_a_ of PCL/SBA3 90/10 and PCL/SBA3_Cu 90/10 (*p* < 0.01) compared to neat PCL. Also, the R_z_ parameter showed the same behavior for all the specimens. Particularly, PCL/SBA3_Cu 90/10 showed lower values of R_z_ than neat PCL (*p* < 0.01) and PCL/SBA3 90/10 (*p* < 0.01).

### 3.4. Contact Angle and Surface Free Energy Evaluation

**Figure 3:**
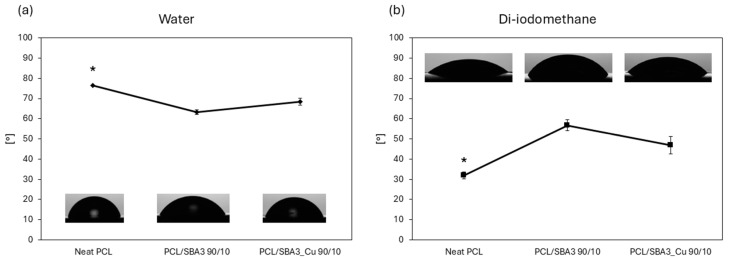
Graphs representing surface contact angle calculated in a hydrophilic environment (water, (**a**)) and in a lipophilic environment (di-iodomethane, (**b**)) (significance: (*) for *p* < 0.05).

A wettability test was performed to evaluate the response of the materials to both hydrophilic and lipophilic environments, as shown in Figure 3. In the hydrophilic environment (Figure 3a), neat PCL exhibited the highest and statistically significant (*p* < 0.05) contact angle (CA) among all samples, with values exceeding 70°, indicating that it was the least hydrophilic substrate. PCL/SBA3_Cu 90/10 showed a CA slightly below 70°, while PCL/SBA3 90/10 demonstrated an even lower CA, suggesting the greatest affinity for the polar solvent. In the lipophilic environment (Figure 3b), neat PCL presented the lowest CA (~30°, *p* < 0.05), indicating the most lipophilic behavior among the materials. PCL/SBA3_Cu 90/10 exhibited a CA of approximately 50°, whereas PCL/SBA3 90/10 had the highest CA (~60°), and therefore the least lipophilic behavior.

The influence of bioactive glass in the polymeric matrix compared to neat PCL is also evident from the calculation of the surface free energy (SFE). Regarding the total surface energy (γ), neat PCL showed the highest value compared to the two composite materials, as shown in Table 5.

### 3.5. Analysis of the Ions in Culture Medium

The concentration of B, Si, and Cu detected in the culture medium after 1, 3, and 7 days of incubation is reported in Table 6, showing a predictable progressive increase with time. The concentration of these elements in the pristine alpha-MEM culture medium is also reported to highlight the effect of incubation time. The concentration of Al is not shown, as it was found always under the limit of detection of the technique for every sample. Differently, the concentration of P and Ca in the alpha-MEM was so high (26 and 62 mg/L, respectively) that it masked the release of these elements from the materials. While the release of B from the two bioactive glasses was slightly higher from PLC/SBA3 than from its copper-doped counterpart, PLC/SBA3 showed a remarkably higher release of Si (up to 62.6 mg/L after 7 days). Finally, Cu was released only from PLC/SB3_Cu, as expected, and it reached significant concentrations in the culture medium (3.39 mg/L).

### 3.6. Protein Adsorption

**Figure 4:**
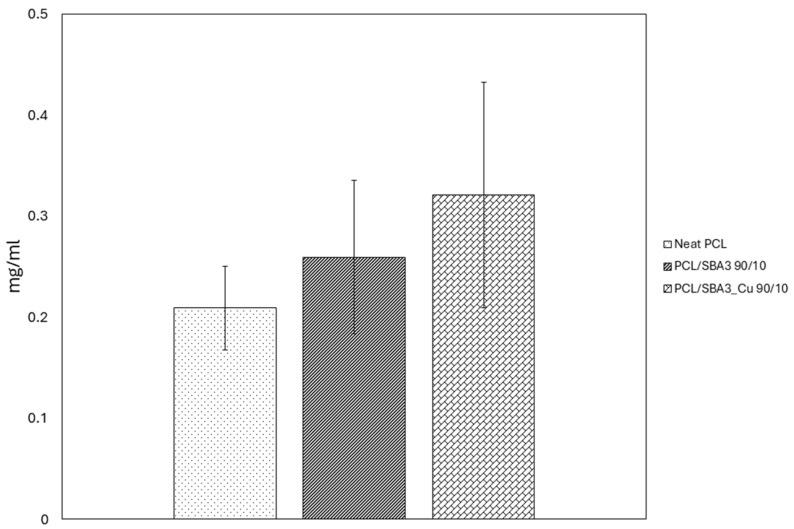
Total amount of BSA protein (mg/mL) adsorbed on the sample surfaces represented as histograms.

The protein adsorption graph (Figure 4) reports the amount of BSA adsorbed on the planar samples of neat PCL, PCL/SBA3 90/10, and PCL/SBA3_Cu 90/10. There was a tendency for the composites to increase adsorption compared to neat PCL, especially the copper-containing one, which showed the highest amount of adsorbed proteins (approximately 0.3 mg/mL) of all the tested conditions, although never in a statistically significant way.

### 3.7. Cell Experiments

#### 3.7.1. Cell Adhesion and Morphology

**Figure 5:**
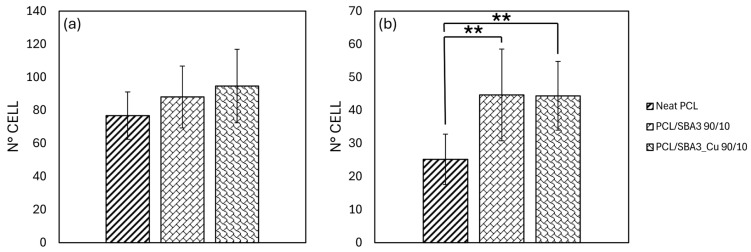
Histograms representing cell adhesion assays of (**a**) ASCs and (**b**) HMEC-1, performed on samples stained with DAPI after 20 min of culture in incubator (significance: ** for *p* < 0.01).

After 20 min, all the specimens allowed the adhesion of both ASCs and HMEC-1 (Figure 5a,b). The number of adherent ASCs increased progressively from neat PCL to SBA3 composite, reaching the highest values with SBA3_Cu, although no statistically significant differences were detected. Indeed, the PCL/SBA3_Cu 90/10 supported the greatest number of adherent ASCs overall, but—even compared to neat PCL—the increase was not relevant (*p* = 0.063). Only with the HMEC-1, PCL/SBA3 90/10, and PCL/SBA3_Cu 90/10 significantly outperformed PCL (*p* < 0.01).

**Figure 6:**
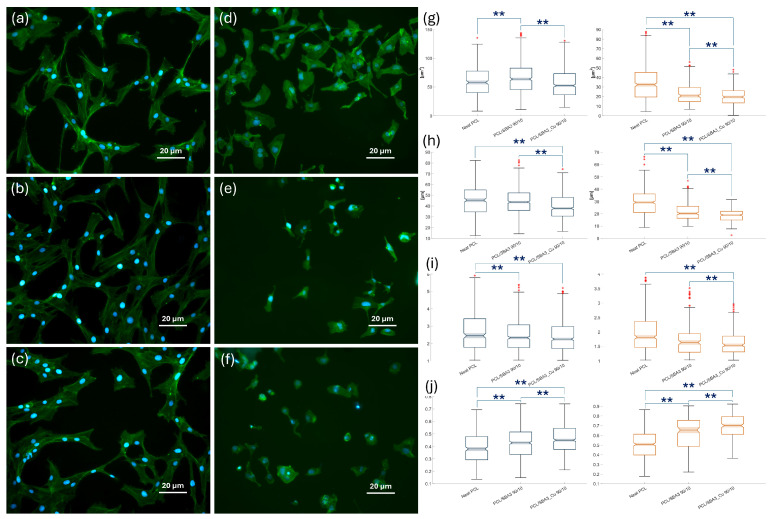
Images and graphs regarding immunofluorescence microscopy acquisition and analysis for cell spreading evaluation, representing ASCs and HMEC-1 on different surfaces, respectively: (**a**) ASCs on neat PCL, (**b**) ASCs on PCL/SBA3 90/10, (**c**) ASCs on PCL/SBA3_Cu 90/10, (**d**) HMEC-1 on neat PCL, (**e**) HMEC-1 on PCL/SBA3 90/10, and (**f**) HMEC-1 on PCL/SBA3_Cu 90/10 after 24 h of incubation (blue color represented the nuclei of cells while the cytoskeleton was stained with green color). (**g**–**j**) represent, respectively, the cellular shape parameters of Area, Perimeter, Aspect Ratio, and Circularity as boxplot (in blue are the ones measured on ASCs, while in orange are those of HMEC-1. Significance: ** for *p* < 0.01).

The analysis of both the cell types spread on specimens was conducted on immunofluorescence microscopy images taken at a common magnification, as shown in Figure 6. On segmented ASCs (Figure 6a–c), the mean area (Figure 6g) values measured for PCL/SBA3 90/10 were significantly higher than those on neat PCL and PCL/SBA3_Cu 90/10 (*p* < 0.01), which reached lower values in the perimeter parameter compared to PCL/SBA3 90/10 (Figure 6h). Consistently, with its highest values overall of perimeter (Figure 6h) and aspect ratio (Figure 6), and the lowest values of circularity (Figure 6j), neat PCL supported more than the two composites both the cellular elongation and the formation of filopodia, as it can be appreciated qualitatively. PCL/SBA3_Cu 90/10 samples were the ones on which, after 24 h of incubation, the ASCs appeared smaller and more rounded in their shape compared to those spread on the neat polymer and PCL/SBA3 90/10, in accordance with the lowest area (Figure 6g), the lowest perimeter (Figure 6h), and the highest roundness (Figure 6j) parameters reported. Only the cell aspect ratio did not differ significantly between PCL/SBA3 90/10 and PCL/SBA3_Cu 90/10 (*p* = 0.152).

As for the HMEC-1 (Figure 6d–f), the shape parameters of mean area, perimeter, and aspect ratio (Figure 6g–i) measured on PCL were, significantly, the highest, (*p* < 0.01 compared to PCL/SBA3_Cu 90/10), while the roundness resulted the lowest (Figure 6j). Conversely, PCL/SBA3_Cu 90/10 expressed the lowest mean area, perimeter, aspect ratio (Figure 6g–i), and the highest roundness (Figure 6j). PCL/SBA3 90/10 was always in between the other two conditions.

#### 3.7.2. Biocompatibility of the Composite PCL/SBA3 Materials

**Figure 7:**
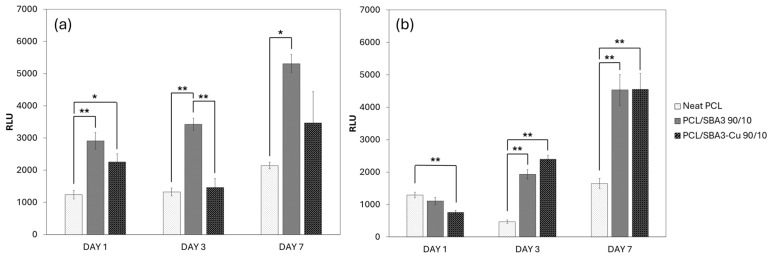
Cell viability after 1, 3, and 7 days measured on each specimen, respectively, for (**a**) ASCs and (**b**) HMEC-1 (significance: * for *p* < 0.05 and ** for *p* < 0.01).

Based on viability tests performed with two different cell types, all the biomaterials were biocompatible, as they sustained cell proliferation over time. More specifically, ASCs (Figure 7a) grew significantly (*p* < 0.05) more on PCL/SBA3 90/10 than on neat PCL at all the time points. This did not occur for PCL/SBA3_Cu 90/10, on which ASCs behaved similarly to neat PCL. Conversely, HMEC-1 cells (Figure 7b) seemingly adapted with difficulty to the neat PCL but responded better and analogously to both PCL/SBA3 90/10 and PCL/SBA3_Cu 90/10, on which they proliferated significantly (*p <* 0.05) more at day 3 and 7 than on neat PCL.

## 4. Discussion

This research aimed at producing PCL-SBA solvent-cast and printed samples suitable for the fabrication of bone substitutes. Based on the authors’ previous experience, a 10 wt.% formulation of filler was chosen to prepare PCL/SBA3 90/10 and PCL/SBA3_Cu 90/10. At the microscopic characterization both the composites exhibited similar surface morphologies, with the filler particles being well dispersed and evenly distributed. However, in PCL/SBA3_Cu 90/10, small filler aggregates were observed, likely due to the higher electrostatic charge of the copper-doped bioglass particles [40]. The EDX spectroscopy confirmed the composition of the specimens according to their content, differentiating, as expected, the SBA3 from the copper-doped bioglass. Regarding PCL, the only detected elements were carbon, nitrogen, and oxygen, as it could be easily anticipated. The percentage of the glass elements (sodium, silicon, and calcium) were detected in higher amounts in PCL/SBA3_Cu 90/10 compared to PCL/SBA3 90/10, likely due to the higher agglomeration and surface exposition, in accordance with Piatti et al. [41]. To better understand the biological effect of PCL/SBA3 90/10 and PCL/SBA3_Cu 90/10, the concentration of significant chemical elements such as phosphorus, calcium, aluminum, boron, silicon, and copper was quantified in the cell culture medium after 1, 3, and 7 days of incubation at 37 °C recurring to ICP-OES. While Al resulted always undetectable, the release of P and Ca was masked by the high concentration of these elements in the alpha-MEM (26 and 62 mg/L, respectively), leaving B, Si, and Cu as the most relevant candidates to induce a biological effect, within the experimental setting adopted.

Under tensile loading, PCL scaffolds exhibited a classical three-phase mechanical response: an initial linear elastic region, followed by a plateau region associated with plastic deformation and a final densification/crystallization phase marked by a steep increase in stress [8]. The elastic modulus of such a PCL scaffold was 51 ± 16 MPa, while the elongation and strength at yield were 7.5 ± 0.8% and 14.3 ± 0.8 MPa, respectively. These parameters depend mainly on print parameters [42,43]. The very high PCL inherent ductility (693 ± 60%) allowed the scaffolds to sustain large strains without brittle failure, which is advantageous for applications requiring compliance and mechanical resilience. The incorporation of SBA3 particles (with and without Cu) resulted in a statistically significant enhancement of E and elongation at yield, while strength at yield and the break parameters decreased only slightly. The increase in elongation at yield may be attributed to several concurrent mechanisms. The inclusion of short, well-dispersed bioactive glass could have promoted stress redistribution within the matrix, thereby delaying the onset of strain localization and macroscopic yielding [44]. Additionally, moderate interfacial bonding between the glass and the PCL matrix could enable mechanisms such as glass pull-out and interfacial sliding, which contribute to energy absorption and strain accommodation before yield [45,46]. These effects collectively resulted in an extension of the elastic–plastic transition, thereby increasing the elongation at yield despite the presence of rigid reinforcements.

It is also important to underline that, despite the incorporation of stiff and brittle glass particles, PCL composites retained the ductile and yielding behavior characteristic of the neat polymer. This can be attributed primarily to the inherent toughness and high elongation at break of the PCL matrix, which continues to dominate the post-yield deformation behavior in composites with low glass content (10 wt%). Furthermore, the glass–matrix interfacial adhesion is often optimized to be neither too weak nor excessively strong. As a result, while the inclusion of bioactive glass significantly increased the composite stiffness, it did not necessarily suppress the matrix ability to undergo plastic flow. This balance of reinforcement and ductility makes these glass-reinforced PCL systems attractive for applications requiring both mechanical strength and compliance, such as load-bearing bioresorbable scaffolds. Moreover, this kind of inclusion of ceramic particles dispersed inside a flexible matrix mimic the real bone structure, even though the tested compounds were not so stiff as real cortical bone structure [47]. The uniform distribution of bioglass in the PCL matrix and the improvement in interfacial adhesion between them, which led to fewer micro-voids [48] were also confirmed by the significant increase in the Vickers hardness.

The incorporation of silica-based bioglass within the PCL matrix slightly decreased the average roughness of the samples, as determined by S_a_, achieving statistical significance only when comparing PCL to PCL/SBA3_Cu 90/10. Overall, the S_a_ values attained, ranging between 0.077 µm (neat PCL) and 0.063 µm (PCL/SBA3_Cu 90/10), can categorize the surface of all the samples as very smooth. More evident differences were detected when considering linear measurements, since R_a_ was between 0.266 µm (neat PCL) and 0.211 µm (PCL/SBA3_Cu 90/10), while R_z_ ranged between 0.157 µm (neat PCL) and 0.055 µm (PCL/SBA3_Cu 90/10), meaning that smoother peaks and valleys distinguished the PCL/SBA3_Cu 90/10 from the neat polymer. Owing to the nature of these parameters, however, careful consideration in reading the data should be encouraged, since R_a_ and R_z_ are calculated, by definition, along a line, while three-dimensional parameters such as S_a_ may provide a more comprehensive assessment of surface roughness by considering variations in all directions. It is also noteworthy that, although such a smooth interface might not be considered the ideal one for a bone substitute material that is clinically used, it is instead very useful for performing in vitro assays aimed at determining the cellular response to the chemical composition of a given material [49].

Based on the contact angle determination, both composites were more hydrophilic (hence less hydrophobic) than the neat PCL, which is consistent with previous reports [50]. The total surface free energy did not differ in a statistically significant way among the samples, ranging between 42.7 mN/m (neat PCL) and 38.8 mN/m (PCL/SBA3_Cu 90/10), while the polar and dispersive components of SE portrayed the behaviors of the materials in the hydrophilic and lipophilic environments. Overall, this was reflected even by the BSA adsorption, which did not differ significantly among the tested samples [51]. Notably, Ca present in bioactive glasses improves the protein adsorption on hydrophilic samples (PCL/SBA3 90/10, PCL/SBA3_Cu 90/10) [52]. As it is known, surfaces with higher surface free energy and better wettability promote cell adhesion and proliferation [53,54,55]. Furthermore, the balance between dispersive and polar components of the surface energy can influence the conformation of proteins adsorbed on surfaces, favoring better cell adhesion, spreading, and subsequent proliferation [56].

The cell response was tested using different assays on two cell types: the ASCs, a mesenchymal stem cell model of osteoblast progenitors [57], and the HMEC-1, representing micro-vessels [58]. These cells are both paramount in colonizing bone substitutes, and their response may give interesting hints for future research. Notably, at the immediate cell adhesion test, which allowed cells to seed for 20 min, ASCs and HMEC-1 showed distinct behavior. The former did not adhere to the substrates in a significantly different way, while the latter preferred PCL/SBA3 90/10 and PCL/SBA3_Cu 90/10 to neat PCL (*p* < 0.01). These adhesion patterns, dependent on the cellular characteristics, were described for various cells and substrates [59].

A morphometric analysis of the two cell lines spread on the specimens after 24 h was conducted on immunofluorescent microscopic images, following established protocols [60]. HMEC-1 cultured on PCL resulted in more spread on the neat polymer than on the composites, showing the highest mean area, perimeter, and aspect ratio, with the lowest roundness. Contrastingly, consistently, the endothelial cells grown on PCL/SBA3_Cu 90/10 expressed the lowest mean area, perimeter, and aspect ratio and the highest roundness. According to the segmentation analysis, on neat PCL, ASCs reached the highest values of perimeter and aspect ratio, and the lowest values of circularity. Together with the fact that the mean area value measured was significantly the highest on PCL/SBA3 90/10 (*p* < 0.01), while the lowest area, the lowest perimeter, and the highest roundness parameters were found on PCL/SBA3_Cu 90/10, these data suggest that neat PCL promoted cellular elongation and filopodia formation in the mesenchymal stem cells more efficiently than the composites.

These observations become more meaningful considering the viability assay performed at 1, 3, and 7 days, even in light of the ion release quantification. The “stress” induced by PCL/SBA3_Cu 90/10 in HMEC-1 after 24 h was completely overcome at the following time points, where the two composites significantly doubled the cell growth sustained by the neat PCL. This is in accordance with the possible role of copper doping in enhancing angiogenesis reported elsewhere [61,62], and it is consistent with an adaptation phase of the cells to the ion-releasing material. The concentration detected in the culture medium after 7 days (3.39 mg/L) was indeed far from the level at which the Cu ions can exert a toxic effect [63]. Quite interestingly, the proliferation curve of the ASCs responded to both the composites, with a clear preference for SBA3 over SBA3_Cu, leading to the hypothesis that these mesenchymal stem cells may be sensitive to B and Si. Specifically, the release of boron from PCL/SBA3 90/10 was more than double at day 1 (0.85 ± 0.06 mg/L vs. 0.36 ± 0.04 mg/L) and still resulted in being slightly higher after one week (2.14 ± 0.06 mg/L vs. 1.63 ± 0.03 mg/L) compared to PCL/SBA3_Cu 90/10. This observation is in accordance with Akdere et al. who tested a wide range of B concentrations (1, 10, and 20 mg/L), and found improvements in cellular proliferation with no toxic effect on ASCs [64]. As for Si, PLC/SBA3 showed an even higher release of this element, which was always at least twice compared to PCL/SBA3_Cu 90/10 (ranging from 46.3 ± 0.3 mg/L vs. 9.5 ± 0.1 mg/L at day 1 up to 62.6 ± 0.2 mg/L vs. 31.2 ± 0.2 after 7 days). The beneficial properties of silicon are well known when dealing with bone substitutes [65,66] and appear consistent with the data of this research. Instead, in the experimental setting adopted, the presence of copper did not ameliorate the optimal performance of SBA3, which doubled the ASCs growth compared to neat PCL since the first day. Within the limits of the study, these results suggest that SBA3 seems more efficient than SBA3_Cu in promoting the proliferation of both mesenchymal stem cells and endothelial cells.

## 5. Conclusions

Further experiments should be performed to investigate whether and how SBA3 and SBA3_Cu can affect osteogenic differentiation of ASCs and vessel-like organization of HMEC-1 at longer culture times with three-dimensional scaffolds, even by exploiting dynamic culture conditions. This advanced experimental setting will help establish the most effective formulation in terms of elemental content and release kinetics, starting from the currently tested bioactive glasses. The percentage of filler load within the PCL matrix should also be a matter of investigation regarding the mechanical features attained. Animal models of large bone defects may be foreseen as the final step, before clinical translation, to assess the angiogenic and osteogenic properties of the selected biomaterials.

## Figures and Tables

**Table 1:** Formulations of the samples.

Sample	PCL (g)	SBA3 (g)	SBA3_Cu (g)
Neat PCL	6	/	/
PCL/SBA3 90/10	5.4	0.6	/
PCL/SBA3_Cu 90/10	5.4	/	0.6

**Table 2:** Standard parameters of water and di-iodomethane.

Liquid	γ (mN/m)	γ^P^ (mN/m)	γ^D^ (mN/m)
Water	72.8	43.7	29.1
Di-iodomethane	50	2.6	47.4

**Table 3:** Mechanical parameters obtained by tensile tests.

Sample	*E* (MPa)	ε_y_ (%)	σ_y_ (MPa)	ε_b_ (%)	σ_b_ (MPa)
Neat PCL	51 ± 16	7.5 ± 0.8	14.3 ± 0.8	693 ± 60	23.4 ± 3.8
PCL/SBA3 90/10	252 ± 35	14.9 ± 3.2	12.7 ± 1.3	647 ± 48	17.8 ± 2.2
PCL/SBA3_Cu 90/10	369 ± 46	14.7 ± 1.5	13.9 ± 1.1	656 ± 47	21.1 ± 1.6

**Table 4:** Profile roughness parameters (R_a_ and R_z_) and Areal texture (S_a_) parameter for the different samples measured with SEM roughness analysis tool.

Sample	R_a_ [µm]	R_z_ [µm]	S_a_ [µm]
Neat PCL	0.266 ± 0.034	0.157 ± 0.029	0.077 ± 0.009
PCL/SBA3 90/10	0.218 ± 0.049	0.074 ± 0.022	0.076 ± 0.015
PCL/SBA3_Cu 90/10	0.211 ± 0.028	0.055 ± 0.006	0.063 ± 0.006

**Table 5:** Total, polar, and dispersive surface energy of samples calculated with the OWRK method.

Sample	Surface Energy: Total [mN/m]	Surface Energy: Polar [mN/m]	Surface Energy: Dispersive [mN/m]
Neat PCL	42.7	2.7	40
PCL/SBA3 90/10	39.2	17.6	21.5
PCL/SBA3_Cu 90/10	38.8	9.9	28.9

**Table 6:** B, Si, and Cu concentration (mg/L) in the culture medium after different time of incubation.

Sample	B [mg/L]	Si [mg/L]	Cu [mg/L]
Alpha-MEM	0.17 ± 0.03	0.23 ± 0.01	N.A.
PCL/SBA3 90/10			
1 day	0.85 ± 0.06	46.3 ± 0.3	N.A.
3 days	1.16 ± 0.03	53.8 ± 0.4	N.A.
7 days	2.14 ± 0.06	62.6 ± 0.2	N.A.
PCL/SBA3_Cu 90/10			
1 day	0.36 ± 0.04	9.5 ± 0.1	1.33 ± 0.01
3 days	0.81 ± 0.03	23.0 ± 0.1	2.58 ± 0.02
7 days	1.63 ± 0.03	31.2 ± 0.2	3.39 ± 0.03

## Data Availability

Data supporting reported results are stored in the computers and laptops of the Bone and Dental Bioengineering Laboratory.
